# Pressure–volume loop-derived cardiac indices during dobutamine stress: a step towards understanding limitations in cardiac output in children with hypoplastic left heart syndrome^☆^

**DOI:** 10.1016/j.ijcard.2016.12.087

**Published:** 2017-03-01

**Authors:** James Wong, Kuberan Pushparajah, Adelaide de Vecchi, Bram Ruijsink, Gerald F. Greil, Tarique Hussain, Reza Razavi

**Affiliations:** Division of Imaging Sciences and Biomedical Engineering, King's College London, St. Thomas' Hospital, London SE1 7EH, United Kingdom

**Keywords:** Magnetic resonance imaging, Congenital heart disease, Hypoplastic left heart syndrome, Catheterisation, Fontan procedure

## Abstract

**Background:**

Children with a single systemic right ventricle, such as in hypoplastic left heart syndrome (HLHS), frequently experience reduced exercise capacity. Elucidating the causes could help with optimising treatment strategies.

**Methods:**

Prospective data from 10 consecutive symptomatic patients with HLHS undergoing clinical cardiac magnetic resonance with catheterisation (XMR) were analysed. Mean age 8.6 years (range 3.5–11.6 years), mean time since Fontan completion 5.5 years. MR-compatible catheters were placed in the systemic right ventricle and branch pulmonary arteries to record pressures at rest, with dobutamine infusion at 10 mcg/kg/min and at 20 mcg/kg/min. Cine short-axis stacks of the ventricle were performed at each condition and used to construct pressure–volume loops.

**Results:**

Compared to rest, cardiac index increased with low-dose dobutamine (*p* < 0.01) with no further rise at peak stress despite a further, albeit, blunted rise in heart rate (*p* = 0.002). A fall in stroke volume occurred (*p* = 0.014) despite good contractility (74% increase, *p* = 0.045) and a well-coupled ventriculo-arterial ratio. End-diastolic pressure and early active relaxation, markers of diastolic function, were normal at rest. However, preload fell at peak stress (*p* < 0.008) while pulmonary vascular resistance (PVR) was low throughout. This group of HLHS patients demonstrated a fall in SV at peak stress, coinciding with a fall in preload.

**Conclusions:**

Markers of systolic and diastolic function remained normal. Failure to adequately fill the ventricle implies a ceiling of maximal flow through the Fontan circuit despite low PVR.

## 1 Background

Despite improvements in life expectancy, patients with single ventricle physiology experience premature circulatory failure [1]. Children with a systemic right ventricle (RV), such as in hypoplastic left heart syndrome (HLHS), have the highest incidence of exercise intolerance [2]. There is evidence that this is related to a limited ability to increase cardiac index (CI) [3]. However, the physiological mechanisms underpinning these findings remain incompletely understood.

Recent work has shown that the application of pharmacological stress in this group of patients results in a fall in end-diastolic volume (EDV) with a blunted cardiac output response. Cardiac output is augmented only through increasing heart rate [4] [5]. Evidence suggests impaired systolic and diastolic function may contribute towards reduced cardiac output [6], [7], but load-dependent measures of function in this group of patients may give inconsistent results [8], [9], [10]. Magnetic resonance imaging combined with catheterisation (XMR) [11], [12] permits invasive measurements of pressure with simultaneous quantification of blood flow and ventricular volume [13]. This assessment offers useful insight into the energetic performance of the RV [14] [15] including the opportunity to construct pressure–volume loops [16], [17] to enable a comprehensive analysis of the physiology through assessment of load-independent indices [15], [18]. However, measuring cardiac function solely under baseline conditions in patients with HLHS may mask dysfunction seen only during exertion. Pharmacological stress therefore offers an alternative method to simulate some of the physiological effects of higher heart rates (HR) in this group of patients [5], [18].

In this study, we assessed a group of children with HLHS exhibiting symptoms of diminished exercise capacity. We focused on assessing ventricular energetics and function while applying incremental dobutamine stress to study the changes in physiology through a range of heart rates. We hypothesized that stepwise increments of dobutamine might unmask ventricular dysfunction only seen at the highest heart rates helping us to further understand the reasons for a blunted response in CI.

## 2 Methods

### 2.1 Study design

**Fig. 1:**
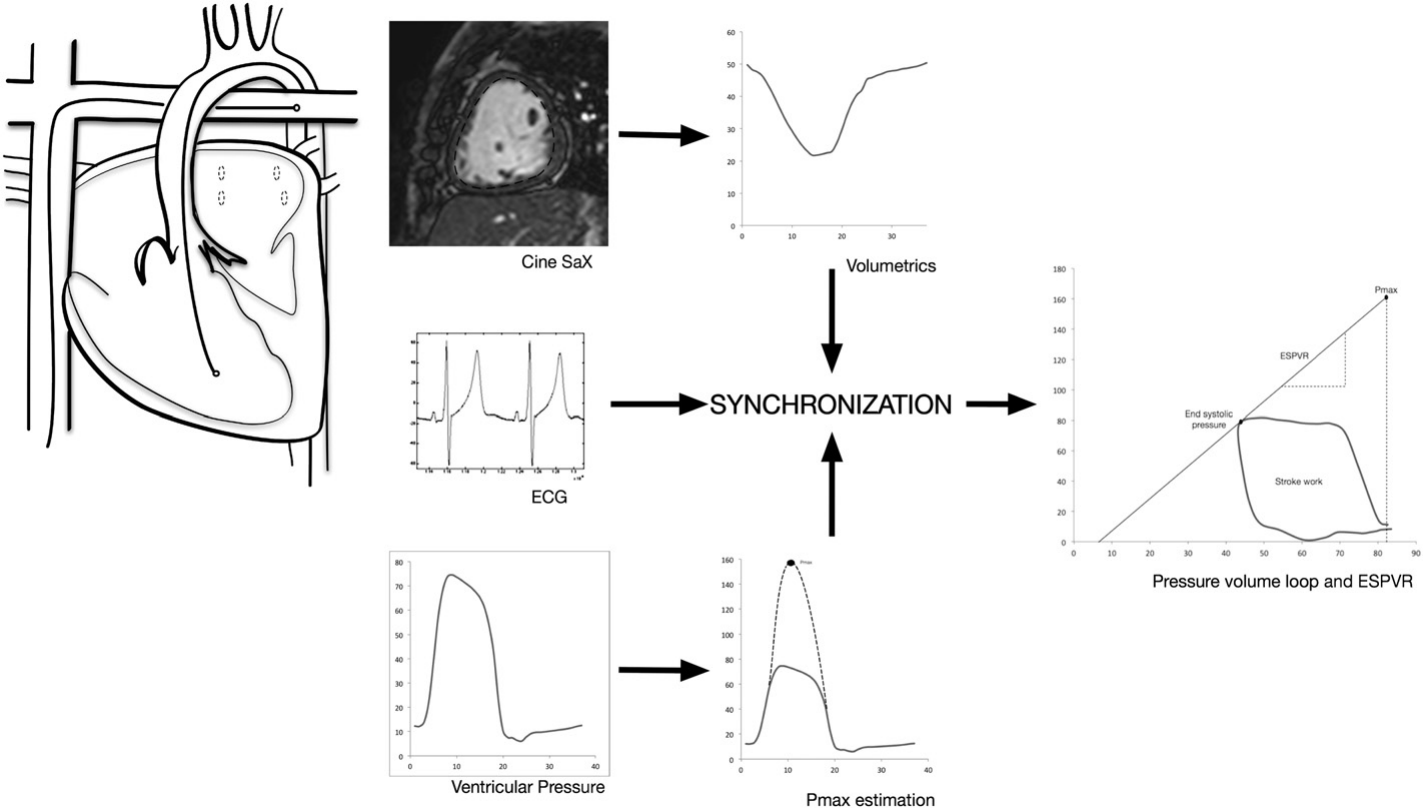
Workflow for composing pressure–volume loops using cine CMR and invasive ventricular pressure measurements. MR-compatible catheters are positioned within the RV and the Fontan circuit. Ventricular pressure traces are synchronised to CMR volumetrics to generate a pressure–volume loop.

Prospective data were collected from 10 consecutive studies on patients with HLHS undergoing clinical XMR. Patients were referred by their clinician for assessment of exercise intolerance. All had undergone completion of a total cavopulmonary anastomosis at our institution and were scanned under general anaesthesia using low-dose inhaled sevofluorane with remifentanyl while maintaining normocarbia as per institutional preference (Ethical approval 09/H0804/62). All subjects underwent cardiac magnetic resonance imaging (MRI) on a 1.5-Tesla scanner (Achieva; Philips Healthcare, Best The Netherlands) using a two-channel coil. The research protocol is outlined in Fig. 1.

### 2.2 Cardiac catheterization

A fluid filled magnetic resonance (MR)–compatible catheter was placed in the systemic RV under X-ray guidance. A second MR-compatible catheter was placed in one of the branch pulmonary arteries. A mean pulmonary artery and mean pulmonary capillary wedge pressure were recorded and the difference used to calculate the trans-pulmonary gradient. Pressures were recorded on a haemodynamic tracer system (TechnoMed Medical Solutions, UK). Patients received a standard heparin bolus for anti-coagulation, which was monitored using activated clotting time. Once the catheters were in-place, the patient was transferred to the MR scanner to undergo simultaneous pressure and volume measurements [19].

### 2.3 Cine MRI

A retrospectively ECG-gated balanced-steady state-free precession (bSSFP) cine short-axis stack planned parallel to the atrio-ventricular valve plane of the systemic ventricle was performed. Images were acquired during end expiratory breath-holds covering apex to base. Typical imaging parameters: TR = 3.0–3.6 ms; TE = 1.5–1.8 ms; parallel imaging (SENSE) factor 2; flip angle 60°; field of view 200–400 mm, slice thickness 6–8 mm depending on patient size, in-plane resolution 1.3–2.0 mm; acquired temporal resolution 30–40 phases, breath-hold duration 5–7 s per slice, 10–14 slices to cover the heart.

### 2.4 Two-dimensional phase contrast flow CMR

A free-breathing retrospectively ECG-triggered two-dimensional (2D) phase contrast (PC) flow was acquired orthogonal to the ascending aorta at the level of the right pulmonary artery (RPA) and additionally orthogonal to the superior and inferior vena cavae (SVC and IVC) with NSA 3, spatial resolution 1.5 × 1.5 × 6 mm, acquired temporal resolution 30 phases. This was used to determine aorto-pulmonary collateral (APC) flow.

### 2.5 Pharmacological stress protocol

Invasive ventricular pressures, pulmonary, and pulmonary capillary wedge pressures were recorded at rest and repeated with infusion of dobutamine first at 10 mcg/kg/min and then at 20 mcg/kg/min. Simultaneous cine short-axis stacks of the ventricle with aorta, left pulmonary artery (LPA), and right pulmonary artery (RPA) 2D PC flow were performed during each condition. The protocol was terminated if the patient became haemodynamically unstable (a supra-physiological rise in blood pressure or HR).

### 2.6 Analysis

Analysis of CMR volumetric and flow data was performed using a Viewforum workstation (Viewforum 2.0, Philips Healthcare, Best, Netherlands). Segmentation of the ventricular cavity involved manual tracing of endocardial contours for each slice at all time points. The position of the atrio-ventricular valve was confirmed by linking the short-axis stack to a long-axis view of the ventricle. Calculation of 2D PC flow stroke volume (SV) was performed by contouring a region of interest encompassing the vessel for all phases of the cardiac cycle.

Aorto-pulmonary collateral (APC) flow was calculated as follows:

$$Q_{\mathrm{APC}}=Q_{\mathrm{Ao}}-\left(Q_{\mathrm{SVC}}+Q_{\mathrm{IVC}}\right)$$

where

Q = flow

APC = aorta-pulmonary collateral

Ao = ascending aorta

SVC = superior vena cava

IVC = inferior vena cava

Pressure–volume loops were constructed for each condition. This is shown in Fig. 1. The end-systolic pressure–volume relationship (ESPVR) was derived at each state from a validated maximal pressure (P_max_) estimation method using the average of multiple pressures [20]. The slope of the ESPVR represents the elastance of the ventricle (E_es_). The slope of the relationship of EDV to end-systolic pressure (ESP) defines the elastance of the artery (E_a_). Ventriculo-arterial coupling is represented by the ratio of E_es_ to E_a_. The area within the pressure–volume loop was used to establish ventricular stroke work and multiplied by HR to calculate power. Tau (τ), the early relaxation constant, was calculated from the reciprocal of the natural logarithm of the early maximal fall in ventricular pressure during diastole.

Preload values were taken from multiple averaged measurements and calculated using a modified *La Place* formula [21]:

$$\sigma =\frac{\mathrm{Pr}}{h\left(2+\frac{h}{r}\right)}$$

where

σ = mean end-diastolic wall stress

P = RV pressure (dynes/cm^2^)

r = endocardial radius of curvature (cm)

h = wall thickness (cm)

The branch pulmonary artery flows and trans-pulmonary gradient were used to calculate pulmonary vascular resistance [13].

### 2.7 Statistical analysis

Statistical analysis was performed using SPSS version 22. Tests of normality were performed using the Shapiro–Wilk method. Continuous variables were expressed as mean ± SD. One-way analysis of variance (ANOVA) was used to assess differences between rest and each condition of dobutamine using Bonferroni-adjusted post hoc *t* tests. Throughout, a *p* value of < 0.05 was considered significant. Inter-user variabilities for manual segmentation of CMR were determined by intra-class correlation coefficients using a two-way random model with absolute agreement.

## 3 Results

Ten consecutive studies were performed with all patients having undergone staged palliation for HLHS. All were prospectively recruited and all tolerated the full dobutamine protocol. Table 1 shows the demographics. Mean age, 8.6 years (range 3.5–11.6 years); mean weight, 25.8 kg (range 16–46 kg); mean time since Fontan completion, 5.5 years (range 0.5–9 years). No significant tricuspid regurgitation in any patient. All subjects recruited into the study were referred for assessment of reduced exercise capacity.

### 3.1 MRI-derived indices of cardiac function

Table 2, Table 3 show haemodynamic response and MRI-derived parameters. Ejection fraction (54.0 ± 6.7%) was in the normal range, but baseline CI was low (2.7 ± 0.6 L/min/m^2^) compared to published values for healthy children [22]. During administration of 10 mcg/kg/min dobutamine, there was an expected rise in CI (*p* = 0.01). This was driven solely by a rise in HR (*p* = 0.0001). SV remained the same as although ESV fell (*p* = 0.001), there was a similar fall in EDV (*p* = 0.0001). At maximum stress with 20 mcg/kg/min dobutamine, there was no further increase in CI, HR rose (*p* = 0.002); however, SV fell (*p* = 0.045) as the fall in EDV was greater than the fall in ESV.

### 3.2 Contractility and coupling

**Fig. 2:**
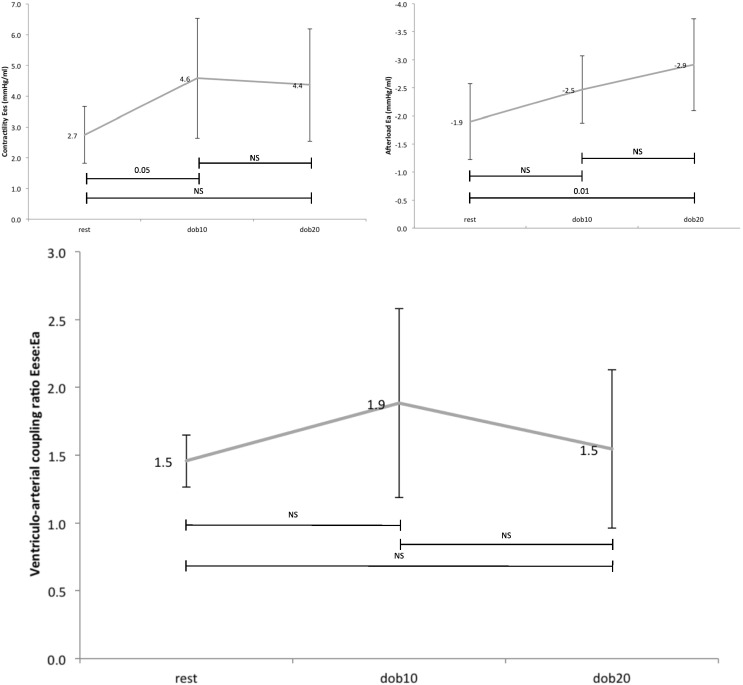
Mean effect of dobutamine stress on contractility (top left), afterload (top right), and ventriculo-arterial coupling ratio (lower).

Fig. 2 shows the XMR-derived indices of contractility and coupling. Contractility (as measured by end-systolic elastance, E_es_) increased 67% during the low-dose dobutamine stress (10 mcg/kg/min) (*p* = 0.045) with no further increase with dobutamine 20 mcg/kg/min. This was accompanied by a stepwise increase in afterload (as measured by arterial elastance, E_a_), which reached significance at dobutamine 20 mcg/kg/min (*p* = 0.012).

The ventriculo-arterial coupling ratio (E_es_:E_a_) was 1.45 ± 0.2 at rest. It remained adequately coupled during administration of incremental doses of dobutamine due to increases in both contractility and afterload.

### 3.3 Ventricular work

**Fig. 3:**
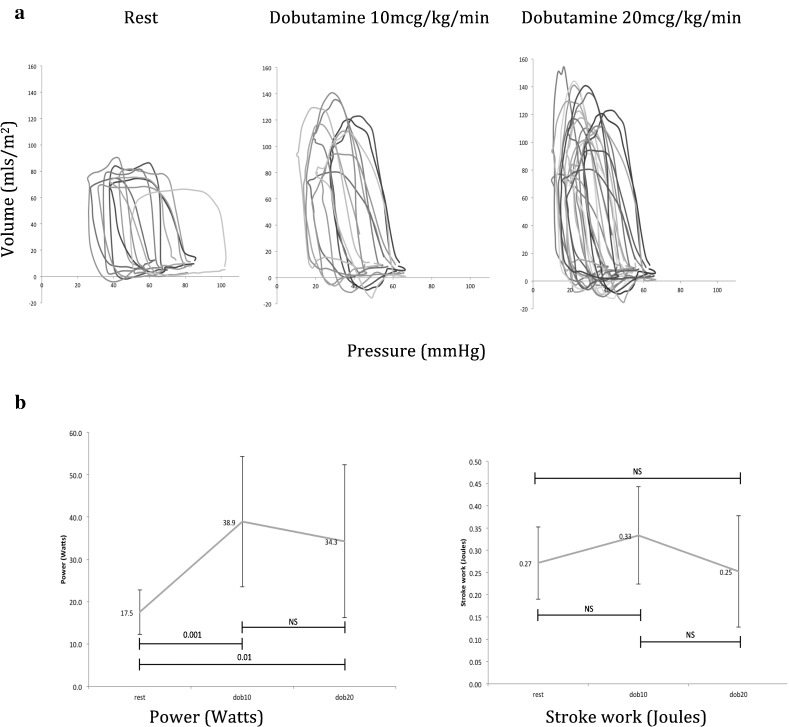
(a) Pressure–volume loops for all of the studies: at rest (left); with dobutamine at 10 mcg/kg/min (middle); and with dobutamine at 20 mcg/kg/min (right). Volumes are indexed to body surface area to allow comparison of loops. (b) Response of power (left) and stroke work (right) to dobutamine.

Fig. 3a shows pressure–volume loops for each condition of dobutamine. During dobutamine stress, although there was a change in the shape of the loop, stroke work did not alter from resting conditions; however, large corresponding increases in HR meant cardiac power increased significantly (114%) with dobutamine 10 mcg/kg/min (*p* = 0.001) but plateaued with dobutamine 20 mcg/kg/min as the late fall in SV offset any further incremental rise in power (Figure 3b).

### 3.4 Myocardial relaxation

Parameters of diastolic function were normal at rest (Table 4) with an end-diastolic pressure (EDP) and early relaxation constant (Tau) that compared to the literature. EDP remained low throughout the study, which is not in keeping with the rise that usually occurs during stress in healthy subjects. When indexing EDP to the RVEDV (mmHg/ml) to give an indexed filling pressure, there was no significant change with application of stress (*p =* 0.99). There was an overall stepwise improvement in myocardial relaxation as demonstrated by the fall in Tau, which reached significance at dobutamine at 20 mcg/kg/min (*p* = 0.038).

### 3.5 Preload and PVR

**Fig. 4:**
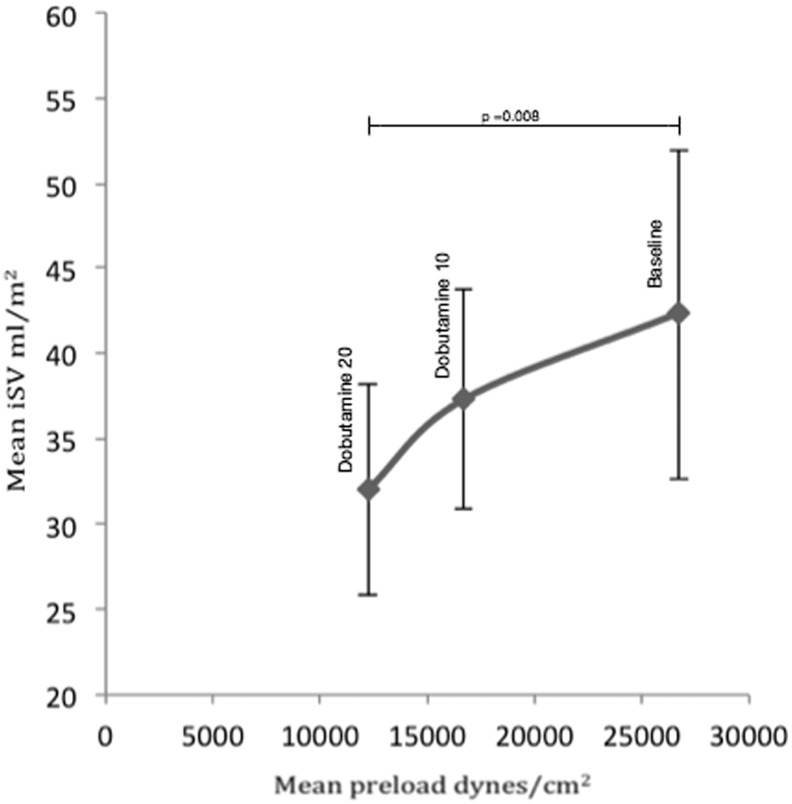
Relationship of mean preload to mean indexed SV. Standard deviation bars are shown. The fall in stroke volume with increasing dobutamine stress is matched by a fall in preload.

There was a stepwise fall in preload, which reached significance with dobutamine at 20 mcg/kg/min (*p* = 0.008). The relationship of preload to SV is shown in Fig. 4. The change in preload was not related to PVR, which was in the normal range (1.7 ± 1.2 WU.m^2^) and remained unchanged throughout (Table 4). APC flow contributed 12.9% of cardiac output at rest (Table 3).

### 3.6 Reproducibility of ventricular volumes and aortic flow

Intra-class correlation (95% confidence interval) was 0.97 (0.80–0.99) for EDV, 0.95 (0.82–0.980) for ESV, 0.95 (0.82–0.98) for SV, and 0.89 (0.58–0.97) for EF.

## 4 Discussion

In this study, we used XMR to gain further insights into the causes of reduced functional status in patients with HLHS post-Fontan. We hypothesised that abnormalities in systolic or diastolic indices of the systemic right ventricle might become apparent with application of stepwise increments of dobutamine stress and these may in part explain the abnormal CI and blunted response to stress previously demonstrated in the literature [4]. However, in this study, using pressure–volume loop-derived indices of function, we did not find any abnormalities in systolic or diastolic ventricular function. In fact, our symptomatic group of patients had normal functional indices and the major factor limiting SV and CI was a fall in preload with a corresponding plateau in indexed EDP.

### 4.1 Relevance to previous studies

Previous studies have shown [4] that CI is low at rest with a blunted response to dobutamine stress compared to normal left ventricles. The rise in CI with dobutamine 10 mcg/kg/min is driven by an increase in HR, and there is no further rise in CI at peak stress [5]. This is because SV does not rise with dobutamine 10 mcg/kg/min and in fact falls with dobutamine 20 mcg/kg/min. This inability to increase SV is driven by an incremental fall in EDV and is unrelated to PVR. The use of XMR allowed us to gain additional insights into the physiology of the blunted CI response in these HLHS post-Fontan patients during dobutamine stress. Dobutamine acts on the adrenergic receptors of the heart and also on peripheral receptors. At low levels, it has mainly inotropic effects on the myocardium with increasing chronotropic effects at higher levels. At high levels, it also reduces peripheral afterload. Dobutamine has been shown to be safe and reliable at levels up to 40 mcg/kg/min [12], [23], [24], [25] [26]. Dobutamine has a logarithmic relationship to the dose response curve. Once the early dose response is achieved, a doubling of the dose leads to a doubling of the haemodynamic effect [12]. In healthy adults, dobutamine increases SV while EDV remains stable. These are similar to the volumetric changes seen during exercise [27]. Studies assessing ventricular function in response to dobutamine [5], [18], [24] and exercise [28] have shown similar findings in patients post-Fontan: a low baseline CI and with moderate stress a HR-driven rise in CI with no change in SV. These studies involved older patients with mixed systemic ventricular morphology. None had looked at high-dose dobutamine, although recently, *Van De Bruaene* et al. [28] showed a similar finding of no additional rise in CI between moderate and peak exercise. The possible causes for the observed fall in SV with peak stress may be [1] inadequate preload, [2] abnormal systolic or diastolic myocardial properties, [3] altered afterload or uncoupling of the heart, or [4] or combination of all of these.

### 4.2 Preload and PVR

In Fontan circulation, the absence of a subpulmonary ventricle might be expected to reduce the preload of the ventricle [29]. This could lead to an inability to respond to the higher systemic venous return needed during stress [30], [31]. In this study, the observed resting preload values for the systemic RV were of a similar magnitude to the healthy LV (32 × 10^3^ ± 4 × 10^3^ dynes/cm^2^) [32]. However, during dobutamine stress changes in preload mirrored SV with an unexpected fall at peak stress. We were able to unmask a limitation in the filling of the ventricle that was not evident at resting conditions.

The observed preload response was not caused by an abnormal PVR. Baseline PVR was normal and remained low throughout the procedure. *Schmitt* et al. used a similar method for quantifying PVR [18] in an older group of Fontan patients. Elevated resting values fell to normal during administration of dobutamine. The difference in PVR between this study and ours is reflected in the difference in ages between the two study groups. Completion of the Fontan results in an absence of pulsatile blood flow. Older subjects have had a longer exposure to decreased levels of endothelin and a higher cumulative risk of pulmonary thromboembolism increasing their likelihood of raised pulmonary tone. This may explain the increased benefits seen with administration of sildenafil in older Fontan patients [28] compared to children during exercise [33]. Interventions to improve the trans-pulmonary gradient are currently focusing on minimising power loss within the Fontan circuit [34] and new techniques to improve the duration of forward flow through the systemic veins may offer therapeutic benefits.

### 4.3 Systolic function

Previous studies using load-dependent markers of function have suggested that systolic function as measured by EF is reduced in those with single systemic RV circulations compared to those with single systemic LV circulations [6]. Our data for EF fall into a similar range as that published in the literature. Interestingly, through the construction of pressure–volume loops, we were able to look at load-independent measures of systolic ventricular function and found contractility increased 67% in response to dobutamine 10 mcg/kg/min while power output increased by 114%. The systemic RV demonstrated good load-independent systolic function albeit at a high energetic cost. The disparity in energetic cost is related to the raising of CI through HR rather than a SV response. Most of the external work performed by the heart is consumed in overcoming systemic afterload. Increased CI mediated by HR causes a disproportionate increase in this work. The increase in HR seen between moderate and peak dobutamine stress was without an increase in CI and so further reduced energy efficiency.

### 4.4 Diastolic function

Findings from echocardiographic-based studies suggest diastolic abnormalities exist in 67–72% of patients undergoing Fontan [6], [7]. However our patient cohort had normal EDP and Tau at rest. These findings are in accordance with studies assessing function using a catheterization technique [18], [24]. Abnormal diastolic myocardial properties are difficult to distinguish from abnormal preload when using load-dependent echocardiographic indices [35], and this may explain the differences in findings. We assessed if diastolic dysfunction was unmasked during stress. Interestingly, throughout dobutamine administration, EDP remained abnormally low. However, Tau, a measure of active early diastolic relaxation, fell, indicating improving, rather than abnormal, ventricular relaxation. Those with HLHS often have a dilated ventricle as a consequence of the previous volume loading effects of a shunt-dependent circulation. To account for this, we indexed EDP to the RVEDV to provide a measure of filling pressure per millilitre of blood ‐there were no significant changes. This lends further support to restricted preload rather than diastolic dysfunction as a cause for reduced cardiac output in this group of patients.

### 4.5 Afterload and coupling

The pressure–volume loops (Figure 3) for the systemic RV displayed a similar pattern to that described in the literature for those with pressure-loaded RVs [36]. During administration of dobutamine, a gradual increase in the afterload was observed. However, the systemic RV matched the increased hydraulic load by increasing E_es_. Under resting conditions, the systemic RV has a ventriculo-arterial coupling ratio that compares favourably to the healthy LV [37] and this remained well matched throughout the application of pharmacological stress indicating good energetic efficiency [38], [39]. The systemic RV displayed good contractile reserve and effective coupling during stress may explain the limited benefit found from administering afterload-reducing agents to this group of patients during exercise [40].

### 4.6 Aorto-pulmonary collaterals

Schmitt and colleagues [18] demonstrated the contribution of mean APC flow on cardiac output doubles during stress. We quantified APC contribution only during resting conditions due to the length of the study. Our baseline values were of similar magnitude and we would expect them to increase in the same way. APC flow has the benefit of contributing to preload but leads to diversion of blood from systemic perfusion. Despite this contribution, it was unable to offset a fall in preload and EDP under peak stress conditions.

## 5 Limitations

In those with a Fontan circulation, peripheral muscle contractions and respiratory effort play a prominent role in driving blood through the systemic veins [41]. Care must be taken interpreting data from anaesthetised patients performing breath-holds. However, the results of this study correspond to those observed by subjects undergoing CPET, namely, a limited CI and fall in SV [3]. In a recent study, assessing the effects of supine cycling on CI in adults with single ventricle circulations [28], SV fell with the highest levels of exertion corresponding to the highest level of dobutamine administered in this study. Pharmacological stress mimics many of the physiological changes the heart is subjected to during exertion and was useful in our patient group who were too small to ride an exercise bike.

The patients in this study were anaesthetised using sevofluorane and remifentanil. We tried to manage the patients with the lowest doses of these drugs to maintain effective anaesthesia. The effects of sevofluorane on loading conditions and contractility have been extensively studied in animals [42]. It demonstrates a dose-dependent effect including decreases in systolic arterial pressure, heart rate, cardiac index, left ventricular minute work index, maximum rate of rise of left ventricular pressure (LV dP/dt), and systemic vascular resistance. There were no effects on stroke volume and left ventricular end-diastolic pressure. High-dose remifentanil has been shown to reduce indexed stroke volume, heart rate, and mean arterial blood pressure [43]. However, the synergistic effects of these two anaesthetic agents appear to alter the loading conditions and contractility on the heart in different ways to their individual actions. A study performed in 2005 by *Chanavaz* [44] used echocardiography in a group of children undergoing anaesthesia to assess ventricular function at baseline with sevofluorane and then reassess the following addition of remifentanil. The addition of remifentanil was found to reduce heart rate, blood pressure, and cardiac index. However, it led to increased systemic vascular resistance with a fall in contractility which is not in keeping with the actions of either drug alone. The effects of these anaesthetic drugs are dose dependent. As we maintained effective anaesthesia with the lowest dose as possible these haemodynamic effects would have been minimised. Indeed, in an earlier study carried out by our group [4], we compared cardiac function in subjects with HLHS-Fontan to a control group of awake healthy adult subjects. There were no significant differences in indexed end-diastolic volume, indexed end-systolic function and indexed stroke volume. This indicates that through the use of low-dose anaesthetic agents and careful management of physiological parameters we may have been able to alleviate some of the published haemodynamic effects of higher dose general anaesthesia during our study protocol.

The systemic RV is not a true prolate ellipsoid. Although other formulas for preload exist specific to the RV, in HLHS, the septal wall is thought of belonging to the systemic RV so a modified formula for idiopathic hypertension is not applicable [45]. Stress represents a mean value across the entire RV wall, with the potential to underestimate stress on the endocardial layer and in regional areas where the myocardium may be thicker (commonly the septal wall in those with a residual LV). As a comparative estimation, it broadly serves its purpose.

The single-beat estimation method was originally conceived for the LV and has been validated in the RV. Early work on the systemic RV has been performed [18], [46] and our results are in keeping with these findings. Altering preload would have offered a load-independent measure of diastolic function, the end-diastolic-pressure–volume relationship. However, acquiring MRI volumetric data within a suitable timeframe is not yet feasible. Tau was used instead although it primarily assesses early diastolic relaxation.

## 6 Conclusions

Children with HLHS-Fontan display a blunted response in CI and a fall in SV at peak stress due to a failure to adequately preload the heart. The HR-driven response makes this an energetically costly process. PVR remains low, systolic and diastolic functional indices tend to be normal, and the ventricle is well coupled to the hydraulic load. In young patients introducing measures that focus on improving the preload of the heart may offer the most benefits.

## Funding

The Division of Imaging Sciences receives support as the Centre of Excellence in Medical Engineering (funded by the Wellcome Trust and EPSRC; grant number WT 088641/Z/09/Z) and the BHF Centre of Excellence (British Heart Foundation award RE/08/03).

The authors acknowledge financial support from the Department of Health via the National Institute for Health Research (NIHR) comprehensive Biomedical Research Centre award to Guy's & St Thomas' NHS Foundation Trust in partnership with King's College London and King's College Hospital NHS Foundation Trust.

## Disclosures

None.

## Figures and Tables

**Table 1:** Patient demographics.

ID & morphology	Age (y)	Weight (kg)	Time since Fontan (y)	Saturations (%)	TR
1. HLHS	9.2	26.5	5.7	98	Mild
2. HLHS	11.6	45.9	8.9	95	None
3. HLHS	9.2	20	6.7	92	None
4. HLHS	5.9	21.5	3.5	95	None
5. HLHS	5.8	16	3.5	93	Mild
6. HLHS	9.1	14.1	6.5	98	Mild
7. HLHS	5.5	16	0.5	93	Mild
8. HLHS	7.6	30	4.2	92	None
9. HLHS	10.1	34.4	8	93	Mild
10. HLHS	12.2	34.6	6.5	96	None
Mean (± SD)	8.6 (± 2.3)	25.8 (± 10.2)	5.5 (± 2.6)	95 (± 2)	

HLHS indicates hypoplastic left heart syndrome.

TR indicates tricuspid regurgitation.

**Table 2:** Haemodynamic response.

ID	Heart rate (bpm)			iCO (l/min/m^2^)			EF %			Mean BP (mmHg)		
	Rest	Dob10	Dob20	Rest	Dob10	Dob20	Rest	Dob10	Dob20	Rest	Dob10	Dob20
1	53	113	127	2.2	5.0	4.7	49	66	63	46	66	60
2	69	107	142	2.3	3.9	4.3	49	65	67	71	122	120
3	59	95	140	2.1	4.1	4.1	57	81	73	59	110	153
4	76	115	138	2.4	3.9	4.3	46	55	60	59	104	99
5	60	128	141	3.0	4.9	5.3	56	58	66	46	60	73
6	68	95	105	3.3	4.1	4.4	61	70	71	44	56	57
7	65	120	132	3.9	4.4	4.6	67	69	76	41	43	48
8	62	113	141	2.9	4.4	3.4	49	65	55	51	91	81
9	68	141	149	2.5	4.1	4.4	49	68	64	55	71	66
10	69	122	132	2.3	3.1	3.0	57	62	66	42	80	81
Mean	65	115*	135 *	2.7	4.2 *	4.2 *	54.0	65.9 *	66.1 *	51	80 †	84 †
± SD	(± 7)	(± 14)	(± 12)	(± 0.6)	(± 0.4)	(± 0.6)	(± 6.7)	(± 7.1)	(± 6.2)	(± 10)	(± 26)	(± 32)
ANOVA *p* value	**0.0001**			**0.0001**			**0.001**				**0.012**	
Post-hoc compared to rest				* denotes *p* < 0.01			† denotes *p* < 0.05					

bpm indicates beats per minute; iCO, indexed cardiac output; EF, ejection fraction; BP, blood pressure; Dob, dobutamine.

**Table 3:** MRI-derived volumetric indices.

ID	iRVEDV (ml/m^2^)			iRVESV (ml/m^2^)			iSV (ml/m^2^)			APC as % of CO
	Rest	Dob10	Dob20	Rest	Dob10	Dob20	Rest	Dob10	Dob20	Rest
1	80	65	58	38	20	21	41	45	37	14.5
2	65	53	42	31	17	13	33	35	30	10.8
3	63	53	40	26	10	10	36	43	29	27.8
4	69	60	50	36	26	19	33	34	31	22.4
5	80	63	49	30	24	18	49	39	37	12.5
6	81	61	54	31	17	18	51	43	42	0.0
7	72	56	44	12	19	9	61	39	35	7.2
8	97	60	44	49	21	20	48	39	24	21.4
9	74	45	46	37	13	17	37	29	29	6.6
10	58	40	35	25	15	12	33	25	23	6.1
Mean	74	56 *	46 *	32	19 *	16 *	42	37	32 †	12.9
± SD	(± 11)	(± 8)	(± 7)	(± 10)	(± 4)	(± 4)	(± 10)	(± 6)	(± 6)	(± 8.6)
ANOVA *p* value	**0.0001**			**0.0001**			**0.019**			
Post-hoc compared to rest				* denotes *p* < 0.01			† denotes *p* < 0.05			

iRVEDV indicates indexed right ventricular end-diastolic volume; iRVESV, indexed right ventricular systolic volume; iSV, indexed stroke volume; APC aorto-pulmonary collateral flow, CO cardiac output.

**Table 4:** Cardiac diastolic function.

ID	EDP (mmHg)			Tau			Preload × 10^3^ dynes/cm^2^			PVR (WU.m^2^)		
	Rest	Dob10	Dob20	Rest	Dob10	Dob20	Rest	Dob10	Dob20	Rest	Dob10	Dob20
1	8.0	4.8	7.0	28.9	22.8	17.7	80	33	28	0.9	1.7	1.7
2	7.5	9.5	6.0	12.5	6.4	4.3	68	61	27	0.9	0.42	2.0
3	12.5	11.5	10.5	45.7	22.0	18.5	77	53	28	0.8	1.5	1.6
4	4.5	4.5	4.0	28.7	27.0	8.8	37	27	25	2.1	2.5	2.1
5	12.0	9.0	10.7	28.6	12.4	4.6	81	41	36	1.0	0.5	0.5
6	3.5	4.0	6.0	18.0	13.0	6.3	19	18	16	0.9	0.8	0.8
7	10.7	9.5	11.0	62.5	18.8	27.0	89	70	46	0.95	0.9	1.1
8	5.0	2.0	2.0	18.4	18.8	14.2	36	46	36	1.5	1.8	1.7
9	10.0	10.0	7.0	28.8	23.4	19.8	42	31	22	1.9	1.8	1.5
10	6.5	6.3	3.8	22.6	30.7	21.6	32	16	19	4.4	3.7	3.4
Mean	8.1	7.4	7.1	30.1	19.3	13.8 *	52	36	26 †	1.7	1.7	1.8
± SD	± 3.1	± 2.8	(± 2.7)	(14.3)	(7.4)	(8.0)	(30)	(19)	(10)	(± 1.2)	(± 1.1)	(± 0.8)
ANOVA *p* value	0.72			0.005			0.037			0.97		
Post hoc compared to rest				* denotes *p* < 0.01			† denotes *p* < 0.05					

EDP indicates end-diastolic pressure, Tau is the early relaxation constant, PVR pulmonary vascular resistance.
